# What is the meaning of urban liveability for a city in a low-to-middle-income country? Contextualising liveability for Bangkok, Thailand

**DOI:** 10.1186/s12992-019-0484-8

**Published:** 2019-07-30

**Authors:** Amanda Alderton, Melanie Davern, Kornsupha Nitvimol, Iain Butterworth, Carl Higgs, Elizabeth Ryan, Hannah Badland

**Affiliations:** 1 Healthy Liveable Cities Group, Centre for Urban Research, RMIT University, Melbourne, Australia; 2 Bangkok Metropolitan Administration, Bangkok, Thailand; 3 UN Global Compact – Cities Programme, RMIT University, Melbourne, Australia; 4 Department of Health and Human Services, Victoria State Government, Melbourne, Australia; 5 Centre for Health Equity, Melbourne School of Global and Population Health, University of Melbourne, Parkville, Victoria 3010 Australia

**Keywords:** Global south, Health inequities, Indicator, Policy, Sustainable development goals, Urban planning, Urbanisation

## Abstract

**Background:**

Creating ‘liveable’ cities has become a priority for various sectors, including those tasked with improving population health and reducing inequities. Two-thirds of the world’s population will live in cities by 2050, with the most rapid urbanisation in low- and middle-income countries (LMIC). However, there is limited guidance about what constitutes a liveable city from a LMIC perspective, with most of the evidence relating to high-income countries, such as Australia. Existing liveability frameworks include features such as public transport, affordable housing, and public open space; however, these frameworks may not capture all of the liveability considerations for cities in LMIC contexts.

**Objectives:**

This case study formed a multi-sectoral partnership between academics, policymakers (Bangkok Metropolitan Administration, Victorian (Australia) Department of Health and Human Services), and a non-government organisation (UN Global Compact – Cities Programme). This study aimed to: 1) conceptualise and prioritise components of urban liveability within the Bangkok, Thailand context; 2) identify alignment to or divergence from other existing liveability tools; and 3) identify potential indicators and data sources for use within a Pilot Bangkok Liveability Framework.

**Methods:**

The Urban Liveability Workshop involving technical leaders from the Bangkok Metropolitan Administration and a rapid review of liveability literature informed the conceptualisation of liveability for Bangkok. The Bangkok Metropolitan Administration Working Group and key informants in Bangkok provided input into the liveability framework. Indicators identified for Bangkok were mapped onto existing liveability tools, including the UN Global Compact CityScan.

**Results:**

Findings revealed commonalities with the Australian liveability definition, as well as new potential indicators for Bangkok. The resulting Pilot Bangkok Liveability Framework provides a structure for measuring liveability in Bangkok that can be implemented by the Bangkok Metropolitan Administration immediately, pending appropriate data acquisition and licensing. The Bangkok Metropolitan Administration Working Group and key informants identified core issues for implementation, including limited spatial data available at the district-level or lower.

**Conclusions:**

This study conceptualised urban liveability for Bangkok, a city in a LMIC context, with potential for adjustment to other cities. Future work should leverage opportunities for using open source data, building local capacity in spatial data expertise, and knowledge sharing between cities.

**Electronic supplementary material:**

The online version of this article (10.1186/s12992-019-0484-8) contains supplementary material, which is available to authorized users.

## Background

### Global trends: population growth, urbanisation, rise of NCDs and climate change

International agendas such as the Sustainable Development Goals (SDGs), the New Urban Agenda, and the Healthy Cities movement increasingly call for urban settings to promote health and environmental resilience [1–3]. The prioritisation of creating healthy, liveable and sustainable cities responds to an established evidence base supporting the link between cities and health and wellbeing outcomes [4–6] as well as responding to the global trends of rapid population growth and urbanisation. For example, already half of the world’s population live in cities, and an estimated two-thirds of people will be living in urban settlements by 2050, with the most rapid rates of urbanisation occurring in low-to-middle-income countries (LMICs) [7].

At the same time, climate change, widening inequities, globalisation, and the rising burden of non-communicable disease place additional and substantial demands on cities, with these challenges disproportionately affecting LMICs. There is now a pressing need for cities to be ‘resilient’ and mitigate the adverse consequences of these trends [8, 9]. For example, in response to climate change, cities must adapt to new threats, such as rising sea levels, while also working to reduce future greenhouse gas emissions. To be effective, coordinated responses are needed from diverse sectors including government, academia, the private sector, and civil society to create cities and neighbourhoods that are resilient, sustainable, inclusive, equitable, economically productive, and support good health and wellbeing [7].

### Urban liveability and health and wellbeing

In parallel to these global trends, creating ‘liveable’ cities has become a priority for various sectors, including those tasked with improving population health and reducing inequities [10]. Since the beginning of the Healthy Cities movement, there has been increasing recognition of the role of urban environments in shaping human health and wellbeing, prompting calls for urban planning and public health disciplines to reconnect [6, 11]. Urban liveability is closely aligned with the concept of social determinants of health [12] and evidence demonstrates that improving liveability can promote residents’ health and wellbeing while simultaneously reducing a city’s environmental impact. For example, aspects of urban liveability such as public transport [13], neighbourhood walkability [14, 15], and access to quality parks and public open space [16–19] have been positively associated with health outcomes and behaviours, including increased physical activity and improved mental health. These attributes also mitigate the effects of climate change by alleviating the urban heat island effect [20, 21] and reducing car dependence and greenhouse gas emissions [22].

One consideration is that there is limited guidance about what constitutes a liveable city or neighbourhood from a LMIC perspective, with most of the evidence relating to high income country contexts [23, 24]. For example, in the Australian context, liveable cities have been conceptualised as *‘safe, attractive, socially cohesive and inclusive, and environmentally sustainable, with affordable and diverse housing linked to employment, education, public open space, local shops, health and community services, and leisure and cultural opportunities,* via *convenient public transport, walking, and cycling infrastructure’* [25]. However, there are likely other, and / or different prioritisation of, liveability attributes in LMICs that may not be reflected in frameworks developed for cities in high income countries [26]. For example, some residents in LMICs may live in informal settlements, and / or have limited access to clean drinking water and sanitation [27]. These attributes impact the liveability of a city, yet existing definitions of urban liveability do not capture these nuances [12]. Hence, there is a need to contextualise liveability from a LMIC perspective so that actions to enhance urban liveability are responsive to the diverse contexts and aspirations of cities.

Another consideration is whether cities are delivering liveability *for all*, particularly as intra-city disparities in infrastructure provision (e.g. access to reliable public transport) are social determinants of health that translate to health inequities [28, 29]. Indeed, health inequities observed within cities have been highlighted by the WHO as a pressing global issue [30], and the recent Shanghai Declaration calls for stronger city governance and mechanisms that promote greater equity at the local community and city level [31]. Of the numerous liveability indices available, not all of these have been configured to detect inequities in liveability. Rather, some liveability indices are targeted towards assessing the attractiveness of cities for investors or expatriates’ remuneration for relocation; other indices lack the fine-grained spatial scales needed to determine how liveability is distributed across a city [32]. Applying evidence-based indicators at spatial scales smaller than a city is therefore required to identify any potential inequity [32].

One mechanism to address both of these considerations is the development of context specific urban liveability indicators that can be used to measure and monitor progress towards urban liveability [28]. Applying such indicators can stimulate discussion between diverse stakeholders and sectors including policymakers, urban planners, and civil society, while providing information about and prioritising certain social determinants of health across diverse urban environments [28, 33]. Importantly, these indicators must be appropriate to the setting (e.g. LMIC) and sensitive enough to detect disparities in liveability within cities [32].

### Urban liveability frameworks and indicators in LMICs

The Sustainable Development Goals (SDGs) and many aligned tools provide high-level frameworks to guide aspirations for cities globally [10, 34]. The SDGs provide an overarching global framework for enabling and delivering sustainable development [10], and internationally define the scope for the 2030 Agenda for Sustainable Development, which has been ratified by all 193 UN member states. SDG 11 specifically targets urban sustainability, aiming “to make cities safe, resilient and sustainable” [1].

Developed in alignment with the ten universal principles of the United Nations Global Compact in the areas of human rights, labour, environment and anti-corruption, the CityScan diagnostic tool, developed by the Global Compact’s Cities Programme, helps cities identify and rank 157 pressing urban issues in 22 topic areas across the city’s social development, environmental sustainability and governance [35]. Through the UN Global Compact – Cities Programme’s cross-sectoral approach, responses to these challenges are encouraged through a governance framework of municipal government, the private sector, civil society and community [34, 35]. This tool is being further refined to align with the SDGs.

Alongside these global initiatives are the suite of Healthy Liveable Cities Group Liveability Indicators that draw on conceptually derived and empirically tested urban liveability indicators that respond to numerous domains of urban liveability [29, 36–41]. These indicators were developed from a health and wellbeing perspective, with an aim to identify the elements of urban planning and policy that are associated with health (and health inequities) [12, 32].

These tools provide helpful starting points for cities looking to improve health and wellbeing outcomes. Alongside these frameworks, there is a need to understand how urban liveability: is conceptualised in diverse contexts; can be operationalised to track progress towards these aspirations; local definitions and operationalisations align with or diverge from global frameworks.

### Research context: Bangkok, Thailand

Bangkok is the capital of Thailand and has experienced rapid growth and economic development in the past forty years, similar to other cities in LMICs. Bangkok is increasingly home to migrants from other Thai provinces and other Association of Southeast Asian Nations (ASEAN) member countries, with many newcomers drawn to Bangkok’s denser, inner-city areas. This growth has been accompanied by expanding infrastructure and investment in education, health, and technology, yet challenges remain in ensuring equitable access to these key resources and infrastructure. For example, major issues facing the city include concerns about heavy traffic, unhealthy environments and unequal access to high-quality schools. These issues are accompanied by increasing concern about social inequalities, unemployment and insecure work.

There is strong political commitment in Bangkok to increase the city’s liveability and improve residents’ wellbeing, as laid out in strategic planning documents such as the recent 20-year Development Plan for the Bangkok Metropolis. Bangkok’s 20-year Development Plan aims to improve liveability across the city, with special focus on the elderly, residents with disabilities, and those facing disadvantage. The Bangkok Metropolitan Administration has been a key leader in promoting the urban liveability and sustainability agenda in Bangkok, with a focus on how these aspirations can improve the health and wellbeing of all residents.

## Methods

### Origins of the research partnership

This project is underpinned by a partnership between the Bangkok Metropolitan Administration, the UN Global Compact – Cities Programme, the Victorian (Australia) Department of Health and Human Services, and urban scholars at RMIT University (Melbourne, Australia). In May 2017, a group of technical leaders from the Bangkok Metropolitan Administration participated in the Urban Liveability and Resilience Program, a capacity development and training program run by the UN Global Compact – Cities Programme in Melbourne, Australia. This served as an initial catalyst for the development of a partnership between the Bangkok Metropolitan Administration, UN Global Compact – Cities Programme, and Melbourne-based urban scholars and policymakers from RMIT University and the Department of Health and Human Services. It was anticipated that this project would facilitate two-way knowledge sharing between Melbourne- and Bangkok-based partners, whose cities face similar challenges and share common policy goals (e.g. both Melbourne and Bangkok are 100 Resilient Cities member cities).

### Aims and objectives

The aims of this project were to: 1) conceptualise and prioritise components of urban liveability within the Bangkok, Thailand context; 2) identify alignment to or divergence from other existing urban liveability tools, including those used in Melbourne and Australia; and 3) identify potential indicators and data sources for use within a Pilot Bangkok Liveability Framework.

In partnership with the Bangkok Metropolitan Administration, this project sought to accomplish the following objectives:

- to develop a definition of urban liveability suitable for use in the Bangkok context, and potentially other LMICs;
- to establish a Bangkok Metropolitan Administration Liveability Working Group to provide context-specific advice and guidance;
- to identify and prioritise potential liveability indicators aligned to the SDGs and spatial data sources for inclusion in a Pilot Bangkok Liveability Framework;
- to identify core issues for the Bangkok Metropolitan Administration to populate and implement the Pilot Bangkok Liveability Indicator Framework.

**Fig. 1:**
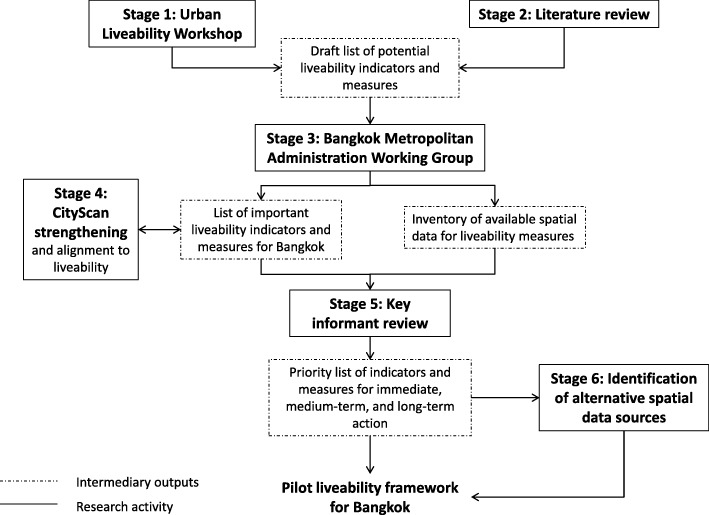
Stages of the research

This project was executed in six stages (Fig. 1). It was purposefully designed as an iterative process to maximise opportunities for Bangkok Metropolitan Administration guidance in terms of maximising relevance to Bangkok’s context and reflecting the Bangkok Metropolitan Administration’s strategic priorities. These methods provide a useful example that can be used by other cities around the world to identify liveability issues and develop indicators. Each stage is discussed in greater detail in the following sections.

### Stage 1: urban liveability workshop

As part of the Urban Liveability and Resilience Program, technical leaders from the Bangkok Metropolitan Administration (Table 1) participated in an urban liveability workshop led by the Cities Programme’s urban scholars and authors of this paper (*Badland, Davern*) [41]. Themes from the Urban Liveability Workshop formed the foundation for the conceptualisation of liveability in Bangkok’s context. In this workshop the Bangkok Metropolitan Administration technical leaders provided insight into similarities and differences in urban liveability for Bangkok compared with Australia, as well as Bangkok’s strategic and priority areas for action. Capacity building and training in using indicators to inform urban planning practice was also embedded in the workshop. The themes discussed by Bangkok Metropolitan Administration provided the foundations for the literature review and Pilot Bangkok Liveability Framework.

**Table 1:** Urban Liveability Workshop participants

Participants at the Urban Liveability Workshop included senior members and technical leads of the Bangkok Metropolitan Administration. Participants represented the following departments: • Strategy and Evaluation Department; divisions included: o Public Health and Environment Strategy o Human Resource and Social Strategy o Administrative Strategy o Infrastructural Strategy o Economic and Financial Strategy o Computer System Control o Computer System Service o Secretarial • Health Department • Fire and Rescue Department • Culture, Sports and Tourism Department • Drainage and Sewerage Department • City Planning Department • Public Works Department • Environment Department • Finance Department • Bangkok Metropolitan Administration Civil Service Commission • Rockefeller 100 Resilient Cities Chief Resilience Officer for Bangkok	

### Stage 2: literature review

A rapid review of international urban liveability literature was undertaken in August 2017 to identify key considerations that may be applicable to the Bangkok context. The scope of the rapid review was defined by the concepts and issues of urban liveability identified by Bangkok Metropolitan Administration leaders, as well as additional considerations for cities in a LMIC context. For example, while drinking water quality was not identified as a salient theme in the Urban Liveability Workshop, the international literature highlights equitable access to high quality, safe drinking water as a key determinant of liveability and health and wellbeing in a LMIC context [10, 42–44].

Combinations of key words capturing the concepts of liveability and LMICs were used in the database Scopus, which was chosen for its multidisciplinary coverage (see Additional File 1 for full search strategy). Qualitative and quantitative empirical literature, theoretical literature, and grey literature were included. The literature search in Scopus yielded 269 results. Screening of titles and abstracts was undertaken to identify potentially relevant articles. Hand searching of relevant articles’ reference lists and of authoritative sources of grey literature (e.g. WHO) was also conducted. Articles were included based on the following inclusion criteria:

- included some discussion, definition, or investigation of liveability in the context of LMICs
- available in full text (online)
- available in English.

Data were extracted from the literature that related to the definitions of liveability, considerations for liveability, and measures or indicators of liveability. These definitions, considerations and measures were grouped into major themes or ‘domains’ of liveability, which were informed by the findings from Stage 1. Together, the findings from Stage 1 and 2 were used to create a draft list of urban liveability indicators for the Bangkok Metropolitan Administration’s consideration. This captured key domains of urban liveability for Bangkok, as well as specific indicators and potential measures that could be used to monitor progress. For example, transport was identified as a key domain of liveability for Bangkok, both in the Urban Liveability Workshop and through the literature review. Within the transport domain, vehicles per kilometre of city roads was a specific indicator that has been used to measure and monitor car congestion in an urban setting [45]. Additional measures were proposed for some indicators based on the project team’s experience in indicator development for Australian cities.

### Stage 3: Bangkok metropolitan administration working group

Scholars from the UN Global Compact – Cities Programme and RMIT University worked with key informants in Bangkok to coordinate and establish a Bangkok Metropolitan Administration Working Group. This working group comprised selected Bangkok Metropolitan Administration technical leaders, including several who had participated in Stage 1 (Urban Liveability and Resilience Program). One of the tasks for the Bangkok Metropolitan Administration Working Group was to review the liveability indicators (generated as part of Stage 3) to ensure the indicators and measures were relevant to the context of Bangkok. The Bangkok Metropolitan Administration Working Group also took an informal inventory of spatial data sources that could potentially be used to measure and monitor liveability in Bangkok.

### Stage 4: City scan strengthening

The urban liveability indicators identified for Bangkok through Stages 1 and 4 were mapped against three existing urban liveability tools: the SDGs [10], the UN CityScan [34], and the Healthy Liveable Cities Group Liveability Indicators. These tools were chosen for their alignment with the social determinants of health and their abilities to influence international and local (Australian) policy. This process took into account the agreement (or disagreement) between high-level indicators, rather than specific measures. For example, the indicator ‘food quality’ that was identified for Bangkok was mapped to the CityScan’s ‘food security’ and the Healthy Liveable Cities Group’s ‘food environment’ indicator. While each of these encompasses a slightly different concept, all three share a common strategic focus on ensuring access to quality food for all residents and achieving at least one target under SDG 2: ‘zero hunger.’

### Stage 5: key informant review

The list of liveability indicators and measures identified in Stage 3 was further refined by Bangkok Metropolitan Administration key informants. The key informants were selected by one of the authors (*Nitvimol*) who was based in the Bangkok Metropolitan Administration. Key informants were Bangkok-based civil servants with a high level of experience and representing a range of government departments involved in the delivery of at least one domain of liveability. The key informants: 1) prioritised liveability indicators and measures for immediate, medium-term, and long-term action by the Bangkok Metropolitan Administration; 2) identified priority measures for each indicator that best captured liveability in Bangkok, taking into account available data sources (where known); and 3) identified data custodians for the priority measures (where known).

The process of prioritising liveability indicators for immediate, medium-term, and long-term action by the Bangkok Metropolitan Administration (Aim 1) considered two main criteria. First, the level of importance of each indicator (as determined by the Bangkok Metropolitan Administration Working Group) was considered. Second, key informants considered the feasibility of measuring each indicator with existing data sources and the timeframes within which these data are/would become available. It was anticipated that this would involve some negotiation in terms of which indicators were determined to be most important; however, in practice, the availability of readily usable spatial data largely determined which indicators were immediately actionable. Hence, there was a high level of consensus during this prioritisation process.

### Stage 6: spatial data sourcing

Where possible, district-level data (or data captured in units smaller than city-level) were identified and incorporated into the framework. The purpose of this was to enable better measurement and monitoring of progress to capture differences and disparities in access to key ‘liveability’ infrastructure within the city of Bangkok, as well as providing a tool to monitor precinct-level developments. Where no spatial data were available in Bangkok for a given indicator identified in Stage 4, alternative potential spatial data sources were suggested for inclusion in the Pilot Bangkok Liveability Framework. These alternative data sources were identified through a desktop review.

## Results

### Aim 1: conceptualise and prioritise components of urban liveability within the Bangkok, Thailand context

Key themes from the workshop revealed strong motives around the SDGs and promoting health and wellbeing for the residents of Bangkok (Table 2). Findings from the workshop also revealed commonalities with the Australian urban liveability definition, as well as some key differences. While the general domains of liveability were similar in the Bangkok and the Australian contexts, the specific indicators and measures for housing differed. For example, housing was identified an important domain in both the Australian and Bangkok contexts. However, in Australia, a major concern is housing stock affordability. In Bangkok, indicators and measures for housing needed to capture informal housing and the impact of flooding on informal housing settlements. The workshop findings also revealed some new indicators of liveability specific to Bangkok’s context. For example, Bangkok Metropolitan Administration technical leaders emphasised the importance of access to temples and cultural opportunities as a core element of social infrastructure in Bangkok, whereas cultural and religious opportunities were not regarded as being as central to social infrastructure in the Australian context.

**Table 2:** Themes from Urban Liveability Workshop

Theme	Liveability concepts discussed in the workshop
Amenity	A safe environment A high level of local amenity (neighbourhood access to services and employment)
Employment	Job security Opportunity to earn a fair wage Equal opportunity Work/life balance (being able to spend time with family and friends) Local employment opportunities
Environmental management	High quality air Zero waste No flooding Greater tree coverage to provide shade Buildings with greater energy efficiency (green buildings) Agile office practices – paperless and access to connected technology
Food	Quality food
Health and wellbeing	Healthy population: both physically and mentally healthy Opportunities for physical activity
Housing	Affordable housing for all
Public open space	Areas for passive recreation and physical activity Green space, pocket parks
Social connectedness	Sense of community and social cohesion around neighbourhoods
Social infrastructure	Access to temples, museums, music and other cultural events that provide opportunities for people to come together Multi-purpose local community centres High quality education and schools
Transport	Reduced/no car congestion Increased provision of transit-oriented developments Connected public transport networks Mass transit availability

Themes from the Urban Liveability Workshop, which aimed to explore concepts of liveability relevant to Bangkok’s context. From Bangkok Metropolitan Administration Report 2017 [41]

### Aim 2: alignment to or divergence from other existing urban liveability tools

As illustrated in Table 3, findings from this stage indicated consistent alignment between the Pilot Bangkok Liveability Framework and the other urban liveability tools examined as part of this project. All of the pilot indicators identified for Bangkok aligned with at least one SDG, with the majority of the indicators supporting multiple SDGs. Further, this revealed key areas of alignment between liveability and CityScan indicators. Each of the pilot indicators for Bangkok supported at least one critical area of the CityScan. As anticipated, the Pilot Bangkok Liveability Framework included indicators that were broadly similar to the Healthy Liveable Cities Group’s liveability indicators, as well as some additional indicators for Bangkok’s context. For example, access to sewerage was identified as an important liveability indicator for Bangkok; however, this indicator is not included in the indicators developed for Australia.

**Table 3:** Pilot liveability indicators for Bangkok were mapped onto existing liveability tools (Sustainable Development Goals, CityScan, Healthy Liveable Cities Group Liveability Indicators)

Urban Liveability Indicators for Bangkok’s Context^a^	SDGs & Relevant International Standards	UN Global Compact: CityScan [35]		Healthy Liveable Cities Group Liveability Indicators [57]	
		Critical Area	Subcategory	Domain	Indicator
Water quality/pollution^b^	SDGs 3, 6, 9, 11, 12, 14	City Sustainability	Water resource management		
Air quality	SDGs 3, 7, 11, 12, 13 WHO air quality targets	City Sustainability	Environmental sustainability	Ambient environment	Air quality
Tree canopy coverage (shade)	SDGs 3, 11, 13, 15 *From 2011 GHD report for City of Melbourne* [46]: target of 30% of city as tree canopy.	City Sustainability	Climate change mitigation		
Flooding	SDGs 1, 3, 9, 11, 13	City Sustainability	Climate change impacts and adaptation		
Drinking water^b^	SDGs 3, 6, 9, 11, 12 WHO drinking water quality targets	City Sustainability	Water resource management		
Waste management	SDGs 9, 11, 12	City Sustainability	Waste		
Sewerage^b^	SDGs 3, 6, 9, 11, 12	City Sustainability	Waste		
Access to fuel^b^	SDGs 7, 9, 11	City Sustainability	Energy		
Food quality	SDGs 2, 3	City Development	Food Security	Walkability	Proximity to supermarkets
Sense of community	SDGs 11	City Development	Social inclusion; Community and culture		
Housing affordability	SDGs 11	City Development	Housing and shelter	Housing	Affordable housing
Local employment opportunities	SDGs 1, 4, 8, 9, 10, 11	City Development	Employment	Employment	Live and work in same SA3
Job security	SDGs 1, 4, 8, 9, 10	City Development	Employment		
Work/life balance	SDGs 1, 4, 8, 10	City Development	Labour Rights		
Opportunity to earn a fair wage	SDGs 1, 4, 8, 9, 10	City Development	Employment		
Mass transit availability; Public transport networks; Transit-oriented developments	SDGs 3, 11, 13	City Sustainability	Mobility	Transport	Proximal access to public transport
Traffic congestion	SDGs 11	City Sustainability	Mobility		
Passive recreation and physical activity locations	SDGs 3, 11, 13, 15	City Sustainability; City Development	Climate change mitigation; Community and culture	Green infrastructure	Size of public open spaces; distance to public open spaces
Green space, pocket parks	SDGs 3, 11, 13, 15	City Sustainability; City Development	Climate change mitigation; Community and culture	Green infrastructure	Size of public open spaces; distance to public open spaces
Access to temples, museums, music and other cultural events; Multi-purpose local community centres	SDGs 11	City Development	Community and culture	Social infrastructure	Culture and leisure (cinema/theatres, museums, art galleries, libraries, community centres)
Safety	SDGs 10, 11, 16	City Development	Public safety		
Education	SDGs 4, 8	City Development	Education	Social infrastructure	Education (state primary schools, state secondary schools)
Health	SDGs 2, 3, 10, 11	City Development	Health and wellbeing	Social infrastructure	Access to health and social services
Local amenity (neighbourhood access to services and employment)	SDGs 8, 9, 11	City Development	Access to employment	Social infrastructure; employment	All (education, sport and recreation, culture and leisure, early years, community centres, health and social services); live and work in same SA3

Key: *SDGs* Sustainable Development Goals, *WHO* World Health Organization, *GHD* GHD Pty Ltd., *SA3* Statistical Area 3 (from Australian Bureau of Statistics)

^a^The liveability indicators for Bangkok were identified through the Urban Liveability Workshop and/or international liveability literature

^b^Indicator was not a salient theme of the Urban Liveability Workshop, but was identified as an important aspect of liveability in the international literature

### Aim 3: potential indicators and data sources for use within the pilot Bangkok Liveability framework

Table 4 shows the Pilot Bangkok Liveability Framework, which was informed by Stages 1–5. The Pilot Bangkok Liveability Framework provides a potential structure for measuring and monitoring liveability in Bangkok that can be implemented by the Bangkok Metropolitan Administration immediately, pending appropriate data acquisition and licensing.

**Table 4:** Priority indicators of liveability for immediate, medium-, and long-term action

Indicator and references	Most useful measure	Data custodian (if known)
Indicators for *immediate* action		
Crime [43–45, 47–50]	Criminal cases per 100,000 persons	Central Information Technology CentreRoyal Thai Police DataNational Statistical Office
Tree coverage [49, 51]	Number of green areas	Department of Environment (Bangkok Metropolitan Administration)
Air quality [43, 45, 49, 52, 53]	Nitrogen dioxide in the air (ppm) Dust/suspended particles in the air – micrograms/m^3^	Department of Environment (Bangkok Metropolitan Administration)
Water quality^a^ [43, 50, 52, 53]	Number of canal water quality testing points showing dissolved oxygen content of ≥2.0 mL/L	Department of Drainage and Sewerage (Bangkok Metropolitan Administration)
Flooding	Number of floods per year	Department of Drainage and Sewerage (Bangkok Metropolitan Administration)
Access to temples [43, 44]	Number of temples per district area	District Office (Bangkok Metropolitan Administration)
Access to schools [44, 50, 54]	Number of schools per 1000 residents (N.B: both primary and secondary schools)	District Office (Bangkok Metropolitan Administration) Department of Education (Bangkok Metropolitan Administration) Ministry of Education
Waste management [44, 45, 51–53]	Average volume (kg) per household of non-recyclable garbage	Department of Environment (Bangkok Metropolitan Administration) District Office (Bangkok Metropolitan Administration)
Indicators for *medium-term* action		
Sense of community [47–49]	Ratio of community population to district population	District Office (Bangkok Metropolitan Administration) Department of Social Development (Bangkok Metropolitan Administration) Strategy and Evaluation Department (Bangkok Metropolitan Administration)
Job security	Unemployment rate	Ministry of Labour The Revenue Department National Statistical Office
Income [44, 45]	Average monthly household income	The Revenue Department National Statistical Office
Education [44, 45]	Percentage of residents with a primary school education	Census
Health [44, 45, 50]	Average life expectancy Number of cases of mental and behavioural disorders	WHO (2016) Health Department (Bangkok Metropolitan Administration)
Local employment [44]	Percentage of residents living and working in the same district	District Office (Bangkok Metropolitan Administration)
Quality food	Percentage of samples of food that is in accordance with health and hygiene standards	Health Department (Bangkok Metropolitan Administration)
Traffic congestion [45]	Number of vehicles per kilometre of city roads	Traffic and Transport Department (Bangkok Metropolitan Administration) Department of Land Transport (BKK)
Sewerage^a^ [42, 45, 52]	Percentage of population with sewerage at their dwelling	Department of Drainage and sewerage (Bangkok Metropolitan Administration) District Office (Bangkok Metropolitan Administration)
Indicators for *long-term* action		
Areas for passive recreation and physical activity [43–45, 47, 50, 51, 55]	Percentage of residents living < 400 m of public open space Percentage of residents living < 400 m of a large park (> 1.5 ha) Percentage of residents living < 400 m of local park	District Office (Bangkok Metropolitan Administration) Department of Environment (Bangkok Metropolitan Administration)
Public transport [44, 45, 50, 51, 55]	Percentage of residents living < 400 m of a local bus stop Percentage of residents living < 800 m of train station	Traffic and Transport Department (Bangkok Metropolitan Administration) District Office (Bangkok Metropolitan Administration)
Housing affordability [44, 45]	Percentage of land being used for informal housing	National Housing Authority Department of Lands District Office (Bangkok Metropolitan Administration)
Work/Life balance	Number of hours of working per day and per week Number of hours per week engaged in leisure activities	Ministry of Labour Ministry of Social development and Human Security Culture Sport and Tourism Department
Access to community centres [44]	Percentage of residents living < 400 m of community centre	District Office (Bangkok Metropolitan Administration) Department of City Planning (Bangkok Metropolitan Administration)
Neighbourhood amenity [44, 51, 54, 55]	Percentage of residents living near locally-defined ‘social infrastructure’ (37)	District Office (Bangkok Metropolitan Administration) Department of City Planning (Bangkok Metropolitan Administration)
Drinking water quality^a^ [42–45]	Percentage of population with piped water	Health Department (Bangkok Metropolitan Administration)
Access to liquefied petroleum gas^a^ [44]	Liquefied petroleum gas connections per household	Ministry of Energy

NB: Within each category (immediate, medium-term, and long-term), indicators are not listed in any particular order. All indicators in this table were first identified by the BMA working group as relevant to Bangkok’s context, then reviewed by BMA key informants for prioritisation into immediate, medium-term, and long-term action. Prioritisation was based on indicator importance for the BMA and the timeframes within which data would become available

^a^Indicator was not a salient theme of the Urban Liveability Workshop, but was identified as an important aspect of liveability in the international literature

The Bangkok Metropolitan Administration Liveability Working Group and key informants identified spatial data issues for populating the indicators. While some promising spatial data were available, limited spatial data available at the district-level or lower were currently available in Bangkok, with most data only available at the city-level or higher. Data custodians were identified for all indicators and measures within the framework. However, feedback from the Bangkok Metropolitan Administration Working Group suggested that further capacity building around the issue of data custodianship and licensing may be required (e.g. building relationships with local data custodians, understanding each custodian’s data processing procedures). Other issues identified included challenges of sourcing or knowledge of available spatial data; including accessing and applying open source data; spatial database architecture and maintenance; and effectively utilising urban liveability indicators to inform evidence-based urban governance and policy decisions.

## Discussion

This research (re)conceptualised urban liveability in the context of Bangkok, a city in a LMIC, using a multi-sectoral partnership. The method provides a great example of how liveability indicators can be used to develop partnerships and build conversations around the multifaceted approaches needed to deal with complex liveability issues across cities. The study was designed to investigate urban liveability using local knowledge alongside the emergent liveability evidence and tools, while ground-testing the pilot framework with various stakeholders through ongoing indicator development, data sourcing, and capacity building. Such an approach enabled the urban liveability framework to reflect the strategic priorities and lived experiences specific to the Bangkok context, which in turn increases the likelihood of translating the framework into policy and practice.

Overall, findings from this study demonstrated points of similarities between the framework developed for Bangkok and other existing liveability tools, while also identifying some key liveability considerations specific to Bangkok’s context. These similarities and differences are discussed in the following section. In the subsequent sections of our discussion, we reflect on future opportunities for Bangkok and other cities, as well as areas for future capacity building in Bangkok.

### Liveability across diverse contexts

The Pilot Bangkok Liveability Framework revealed some similarities between the conceptualisation of urban liveability in Bangkok and in the Australian context. Features such as housing, public transport, public open space, and the quality of the local food environment were conceptualised as being important to urban liveability in both contexts. However, additional considerations for urban liveability were identified for Bangkok, notably: sewerage and solid waste management, quality drinking water, household fuel, informal housing, flooding, and labour rights. Interestingly, these considerations did not feature prominently in a recent liveability index developed for the Khon Kaen district in Thailand [56]; however these differences across the studies may reflect city contexts, stakeholder priorities, and / or project scope. More broadly, the liveability considerations for Bangkok align with those identified in recent liveability frameworks for other cities (including cities in LMICs), such as Pineo and colleagues’ Global Urban Health Index [58] and the Government of India’s recent Liveability Standards [59], all of which reflect the SDGs [10]. In addition, in this study, relationships between several domains of liveability were observed. For example, housing and environmental management domains were both viewed as critical urban liveability domains for mitigating the effects of flooding (health domain). Similar to what others have advocated [6], these findings highlight the complexity of the city as a system and reinforce the need to consider how aspects of urban liveability interact to shape residents’ health and wellbeing and minimise any unintended consequences.

### Implementing the pilot Bangkok Liveability framework: spatial data needs and opportunities for capacity building

This project revealed substantial knowledge of and commitments to the urban liveability agenda and action on the social determinants of health, alongside a willingness to use spatial data in Bangkok. The Pilot Bangkok Liveability Framework represents a significant milestone in the measurement and monitoring of urban liveability in Bangkok, and potentially other cities in LMICs. For Bangkok, it is suggested that measurement of the full suite of liveability indicators (i.e. including those prioritised for medium- and long-term action) is gradually introduced as additional data and resources are sourced.

However, some spatial data challenges likely need overcoming if the proposed framework is to be fully implemented. Spatial data issues that need addressing in future initiatives include generating usable spatial data at scales smaller than a city (e.g. district-level data); others have also pointed to the need for investment in finer-grained data to monitor urban health and wellbeing in LMIC contexts [23, 24]. Open source data, as well as expertise in sourcing and applying such data, could enable the immediate population and measurement of liveability indicators at units smaller than city-level. This would provide a resource-efficient approach to directly measuring implementation of key infrastructure (e.g. public transport) and allow for the monitoring of any disparities in delivery within Bangkok. In addition, Prasad and colleagues have suggested that remote sensing may offer additional opportunities to collect open-source spatial data, while building capacity within LMICs [23]. Other issues that may need addressing prior to implementing the proposed framework include skill development in maintaining spatial databases, negotiating access to spatial data, and developing and applying spatial indicators to support urban planning decision-making.

In light of these core issues, future directions for this work include using an iterative ‘continuous delivery’ approach to sourcing spatial datasets, obtaining the relevant permissions to use these data, calculating and applying the indicators, and building capacity in applying and translating the findings. This process will likely result in further refining the liveability framework presented here.

### Opportunities for reciprocal learning

Cities in high- and low-income country contexts face similar broad challenges as a result of population growth, urbanisation, and climate change; hence, the SDGs call for diverse, multi-stakeholder partnerships, both *across sectors* and *between countries* [10]. (Re)conceptualising liveability for a city in a LMIC sets the foundation for future collaborations and reciprocal learning between cities. For example, working through options for measurement and monitoring that are low-cost, sustainable, and require limited ongoing maintenance (such as open source data) required the research team to be agile, creative, and outward-looking. Further, this study and the (re)conceptualisation of liveability for Bangkok also prompted additional insight about existing liveability tools created for Australia or for global purposes, and a work program run in tandem with this study (CI Butterworth) identified areas for CityScan strengthening (unpublished observations). Finally, the partnership with the Bangkok Metropolitan Administration revealed strong enthusiasm in Bangkok for intersectoral collaboration and working across government departments. Given the calls for more joined-up policy in Australia, continued collaboration with the Bangkok Metropolitan Administration could involve further knowledge sharing around these issues in both contexts.

As these reflections illustrate, there are substantial opportunities for reciprocal learning between diverse cities. Mechanisms and collaborations that encourage further knowledge sharing between diverse cities and contexts are needed to further the progress towards the SDGs. Indeed, the success of achieving the SDGs relies on active and meaningful local, national, and international collaborations [10].

### Limitations

This project should be viewed in the light of its limitations. First, liveability was contextualised from the perspective of Bangkok Metropolitan Administration technical leaders. There may be additional considerations for liveability in Bangkok’s context that should be explored further with a wider range of stakeholders, including civil society, non-government organisations, and advocacy groups. Nevertheless, engagement with the Bangkok Metropolitan Administration, who are the stakeholders that develop and deliver urban planning policy, was also a key strength of this project. Second, as this framework was developed specifically for Bangkok’s context, the results of this project may not be directly replicable or generalisable to other cities. However, this framework and the methods used provide a useful starting point for other cities in LMICs, and could be adjusted for use with input from local stakeholders. Third, while a Pilot Bangkok Liveability Framework has been proposed through this research, it has not been populated and tested. This was beyond the scope of this project. It is likely additional refinements will need to be made to the Framework prior to implementation.

## Conclusion

This project conceptualised urban liveability in the context of Bangkok, a city in a LMIC, with potential for adjustment to other cities. The Pilot Bangkok Liveability Framework provides a future agenda and map for measuring and monitoring liveability in Bangkok with close alignment to the SDGs and social determinants of health. The lack of district-level data for many liveability indicators currently presents a challenge in measuring and monitoring progress towards greater urban liveability in Bangkok. Future work should leverage opportunities for open source data, local capacity building in spatial data expertise, and evidence-based urban governance in Bangkok. This will enable improved monitoring of progress towards achieving greater liveability, and subsequently enhanced health and wellbeing for all through action on the social determinants of health.

## Additional file

Additional file 1: Search strategy (Scopus). Database search strategy used in literature review. (PDF 172 kb)

## Data Availability

Data sharing is not applicable to this article as no datasets were generated or analysed during the current study.
