# Supplementary material for: What is the meaning of urban liveability for a city in a low-to-middle-income country? Contextualising liveability for Bangkok, Thailand
Source: Global Health. 2019 Jul 30;15:51. doi: 10.1186/s12992-019-0484-8 (PMC6668125; doi:10.1186/s12992-019-0484-8)
Supplement: Supplementary file 1 — Search strategy (Scopus). Database search strategy used in literature review. (PDF 172 kb) [file 12992_2019_484_MOESM1_ESM.pdf]

Additional file 1: Search strategy (Scopus)

Export date: 11 Aug 2017

Search:

(TITLE-ABS-KEY(liveab* OR livab*) AND TITLE-ABS-KEY("low income countr*" OR "middle income countr*" OR lmic* OR "low-to-middle-income" OR "low and middle income" OR "developing countr*" OR "less developed countr*" OR "global South" OR "emerg* econom*" OR international* OR Thailand OR Bangkok OR "low income cit*" OR "middle income cit*" OR "urbani?ing cit*" OR "urbani?ing countr*"))
